# Apple Disease Recognition Based on Convolutional Neural Networks With Modified Softmax

**DOI:** 10.3389/fpls.2022.820146

**Published:** 2022-05-03

**Authors:** Ping Li, Rongzhi Jing, Xiaoli Shi

**Affiliations:** School of Electronic Information Engineering, Zhengzhou SIAS University, Zhengzhou, China

**Keywords:** crop disease recognition, modified Softmax classifier, convolutional neural networks, modified convolutional neural networks, disease module identification

## Abstract

Accurate and rapid identification of apple diseases is the basis for preventing and treating the apple diseases, and is very significant for assessing disease disaster. Apple disease recognition from its diseased leaf images is one of the interesting research areas in computer and agriculture field. An apple disease recognition method is proposed based on modified convolutional neural networks (MCNN). In MCNN, Inception is introduced into MCNN, global average pooling (GAP) operator is employed instead of several fully connected layers to speedup training model, and modified Softmax classifier is used in the output layer to improve the recognition performance. The modified Softmax classifier uses the modified linear element as the activation function in the hidden layer and adds the local response normalization in MCNN to avoid the gradient disappearance problem effectively. A series of experiments are conducted on two kinds of apple disease image datasets. The results show the feasibility of the algorithm.

## Introduction

Crop diseases seriously affect the yield and quality of crops and decrease the economic income of the farmers. Because of the various diseases, it is difficult to diagnose the crop disease type cannot in time by the classical disease recognition methods, leading to more and more serious crop disease. Therefore, it is an essential research topic to recognize crop diseases by detecting the disease symptom as soon as they appear on the diseased leaves (Gavhale and Ujwalla, 2014). For crop disease management, it is important to automatically detect and identify the crop diseases at a stage so as to treat them properly. Sometimes farmers are unable to pay attention to the diseases or face difficulty in identifying the diseases (Barbedo, 2013; Zhong and Zhao, 2020). Then, the best chance for disease control may be lost. Every disease has a different remedy to work out. For example, fungi-based disease can be prevented by disrupting the life cycle of the pathogen (Gui et al., 2015). The current approach of disease detection is manual, which means farmers mainly depend on the guide books or use their experiences to identify the diseases (Barbedo, 2013). Martinelli et al. (2015) reviewed the innovative approaches currently under development. Khairnar and Dagade (2014) reviewed a lot of plant disease detection and diagnosis methods, introduced several different feature extraction techniques for extracting various features from plant diseased leaf images. Gulhane and Gurjar (2011) proposed a cotton leaf disease recognition method based on the actual infected image and back propagation neural networks (BPNN). Bashish et al. (2011) proposed a leaf disease detection and recognition method based on K-means-based segmentation and neural networks-based classification. Wang et al. (2012) presented a plant disease recognition method based on principal component analysis (PCA) and several neural networks classifiers by extracting 21 color features, four shape features, and 25 texture features from wheat and grape diseased leaf images, respectively. Fang and Ramasamy (2015) reviewed the direct and indirect disease identification methods currently used in plant disease detection and provided a comprehensive overview of biosensors based on highly selective bio-recognition elements. Sarayloo and Asemani (2015) designed a classifier for fungal disease detection of wheat by pattern recognition techniques. The common steps of the above methods are to extract disease specific image characteristics, and to classify images through a specific classifier, their effectiveness depends on whether the features of artificial selection are reasonable or not, and it is difficult to extract optimal features due to the complex height of crop disease images. Their disadvantages are that image pretreatment process being relatively complex, poor anti-interference ability and robustness, processing speed slow, and generalization ability weak.

Deep learning is a new research direction in the field of machine learning, has been widely applied to various tasks such as computer vision, image classification, target detection, video data analysis, speech recognition, and multimedia retrieval, and achieved excellent results (Brunetti et al., 2018; Rikiya et al., 2018). Deep learning has a deep structure with multi-layer nonlinear mapping. It can transform the feature representation of the image from the original space into a new feature space by transforming the original image, making automatically learn to obtain a hierarchical feature representation, and Conducive to the characteristics of classification, overcoming the inadequacy of manual extraction of specific features in traditional crop disease identification methods. Deep learning provides a new idea for the study of disease identification methods based on crop disease images. Convolutional neural networks (CNN) is a typical deep learning model that has been widely used in many areas. Kamilaris et al. (2018) performed a survey of 40 research efforts that employ deep learning techniques, applied to various agricultural and food production challenges, examined the particular agricultural problems under study, the specific models and frameworks employed, the sources, nature, and preprocessing of data used, and the overall performance achieved according to the metrics used at each work under study, and studied comparisons of deep learning with other existing popular techniques, in respect to differences in classification or regression performance. Their findings indicate that deep learning provides high accuracy, outperforming existing commonly used image processing techniques. Jg et al. (2021) collected 500 field RGB images in a set of diverse potato genotypes with different disease severity (0%–70%) resulting in 2100 cropped images and tested the developed model on the remaining 250 cropped images. The results show that the values for intersection over union of the class background and disease lesion in the test dataset were 0.996 and 0.386, respectively. Qi et al. (2021) proposed a deep learning classification architecture for hyperspectral images by combining 2D convolutional neural network (2D-CNN) and 3D-CNN with deep cooperative attention networks (PLB-2D-3D-A), where 2D-CNN and 3D-CNN are used to extract rich spectral space features, and then, the attention mechanism AttentionBlock and SEResNet are used to emphasize the salient features in the feature maps and increase the generalization ability of the model.

The classical model structure of CNN is closer to the actual biological neural networks, and it has unique advantages in many aspects such as speech recognition and image recognition. It is well known that CNN omits a lot of image preprocessing and processing, and integrates the processes of feature extraction and image recognition by several convolutional and pooling layers and a Softmax classifier. CNN is mainly used to classify the two-dimensional images with invariable scaling, displacement, distortion, and the original image can be directly used as input data without a lot of complex preprocessing in the classical image classification. Dhaka et al. (2021) surveyed the existing literature in applying deep CNN to predict plant diseases from leaf images, presented an exemplary comparison of the preprocessing techniques, CNN models, frameworks, and optimization techniques applied to detect and classify plant diseases using leaf images as a data set, and highlighted the advantages and disadvantages of different techniques and models developed in the field of identification and classification of plant leaf diseases. Kundu et al. (2021) developed a framework of automatic and intelligent data collector and classifier by integrating IoT and deep learning, and designed a “Custom-Net” model as a part of this research. Furthermore, they tested the impact of transfer learning on the “Custom-Net” and state-of-the-art models such as Inception ResNet-V2, Inception-V3, ResNet-50, VGG-16, and VGG-19. Based on the experimental results, they observed that the “Custom-Net” extracts the relevant features and the transfer learning improves the extraction of relevant features. Yadav et al. (2020) proposed a CNN-based recognition method of apple leaf diseases. They applied the contrast stretching based preprocessing approach and fuzzy C-means (FCM) clustering algorithm to segment the disease leaf images, utilized CNN to recognized apple leaf diseases, and validated the performance of the proposed model using 400 image samples (200 healthy, 200) of leaves.

Practice shows that CNN and its improved models have made significant effect in crop disease image processing, and the deeper the CNN model is, the stronger the expression ability of the extracted features is. A very fatal weakness of the traditional CNN is that it has a large number of parameters needed to train, where its fully connected layers have the large number of parameters, about 80% of the parameters of the whole network model. The traditional CNN often face the problem of vanishing gradients during the training, where the fully connected layers are used to stretch the feature map obtained by convolution of the last layer into a vector, multiply this vector, and finally reduce its dimension, and then input it into Softmax layer to get the score of each corresponding category. Problems of full connection: the number of parameters is too large, which reduces the speed of training, and it is easy to overfitting. During the adjustment of parameters, the gradient of the loss function may approach zero, which makes the network difficult. Some network models with excellent performance, such as ResNet and GoogLeNet, replace the fully connected layer with global Average pooling (GAP) to integrate the learned depth features (Hsiao et al., 2019), and GAP itself does not have learnable parameters, reducing a large number of parameters in the fully connected layers, then greatly reducing the number of network parameters, making the model more robust, and avoiding overfitting. In addition, GAP can directly eliminate the features of black boxes in the fully connected layers and achieve any image size input. CNN often uses Softmax as a classifier. It is found that Softmax will converge quickly in the training, and the accuracy of the training will soon achieve 0.9. However, after continuing the training, the accuracy of the test set will improve slowly. If the training is excessive, there will be a decline, i.e., overfitting. Softmax’s loss function is its activation function with the cross entropy, which converts features into probabilities through linear combinations and then uses the cross entropy to calculate losses based on logarithmic likelihood, which leads to poor training results. Many improvements to Softmax have been made to address these issues (Maharjan et al., 2019). The convolutional layer is the core of the convolutional neural network. The convolutional kernel performs operations with the input image through local connection and weight sharing to realize feature learning. Generally, a scale convolutional kernel is used to extract the underlying feature from the input image. By introducing multi-scale convolution kernels, the network can enhance the robustness of features at different scales and achieve high-precision recognition of multi-scale targets. Meanwhile, the network can maintain a fast running speed because no additional parameters and calculations are introduced (Lv et al., 2020). Wang and Zhang (2019) constructed three CNNs of different sizes for the same image, and different convolutional kernels and pooling sizes were used for different networks to obtain efficient feature maps. Inception is a module in GoogleNet and has been validated to be better in the complex images classification tasks. It has multi-scale convolution kernels to extract the features of different scales from the input images by increasing the number of convolutional kernels and introducing multi-scale convolutional kernels. Inception structure has been improved in terms of speed and accuracy.

Inspired by the advantages of GAP, Inception, and modified Softmax, a modified CNN (MCNN) model is constructed for the crop disease recognition problem and is validated and achieved good recognition results. The main contributions of this work are as follows.

1. Inception is introduced into CNN with multi-scale convolutional kernels in different branches of Inception, and multi-scale image features are extracted by different receptive fields in each branch, which increases the width of the network and the adaptability of the network to disease lesion scale.
2. A GAP is adopted instead of the fully connected layers to greatly reduce the number of network parameters, overcome the overfitting problem, and enhance robustness of the model.
3. A modified Softmax is used to classify in the output layer by adopting the modified linear unit as the activation function in the hidden layer and adding the local response normalization processing in the network to effectively avoid the problem of gradient disappearance.

The remainder of the paper is organized as follows. Section 2 reviews the related works including CNN, Inception module, Softmax, and modified Softmax. MCNN is introduced in detail for crop disease recognition in “Modified CNN for Crop Disease Recognition” section. Experiments are presented in “Experimental results and analysis” section. Section 5 concludes the paper and puts forward the future research direction.

## Related Works

### Convolutional Neural Networks

**Figure 1:**
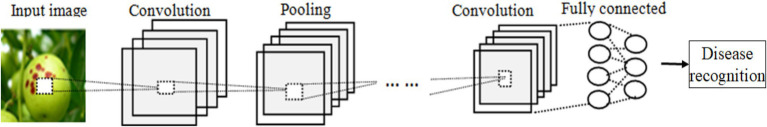
Basic architecture of convolutional neural networks (CNN).

The basic structure of CNN is shown in Figure 1, which consists of an input layer, multiple convolution layers, several pooling layers (or called as down-sampling layers), one to three fully connected layers, and a classification layer, and each layer consists of a lot of independent neurons.

LeNet5 is one of the simple lightweight CNN models (Zhang et al., 2020). It contains two convolution layers, two max-pooling layers, two fully connected layers, and one ReLU layer and one Softmax layer. The main processes of LeNet5 are described as follows:

#### Convolution

The convolutional layer is used to extract feature maps from the input images, the input of the first convolutional layer is the original images, and the input of the subsequent convolutional layer is derived from the pooling layer or the last convolutional layer. The output *x^l^* of the *l*-th layer in CNN is calculated as follows,


(1) $$x^{l}=f\left(x^{l-1}⊗W^{l}+b^{l}\right)$$

where $x^{l-1}$ is output in (*l*-1)-th and t $W^{l}$ is weigh, f(⋅) is an activation function, ⊗ is convolutional operation, and $b^{l}$ is an additive bias in the convolution layer, in default f(x)=1/[1+exp(−x)] .

#### Pooling

Pooling layers often follow convolution layers for down-sampling so as to reduce the computational cost. There are two pooling operators, i.e., average and maximum pooling.

An average (maximum) pooling layer performs down-sampling by dividing the input into rectangular pooling regions and computing the average (maximum) values of each region. A pooling layer takes an h×w×n input and performs the average (maximum) pooling operation with a small pooling size *m* × *m* to produce the so-called pooled maps a $\frac{h}{m}\times \frac{w}{m}\times n$ sub-sampled output. Pooling operation can reduce the computing time of next layers, make the network less prone to overfitting, and extract the invariance features to translation. Suppose there are *N* input maps to a pooling layer, there will be exactly *N* output maps, while the size of the output maps will be smaller. The output of the *l*-th pooling layer can be represented as,


(2) $$x^{l}=f\left(\mathrm{down}\left(x^{l-1}\right)+b^{l}\right)$$

where $x^{l-1}$ is the input of the *l*-th layer, down(⋅) is a sub-sampling function to shrink a feature map by using a pooling operation, and $b^{l}$ is a bias of the *l*-th pooling layer.

#### Dropout

Dropout is a simple and powerful regularization method for CNN to reduce the risk of overfitting by deactivating some randomly selected neurons with a certain probability during the training phase, and forcing the rest neurons to make up for the dropped out neurons, which can make the model more generalized, because it will not rely too much on some local characteristics.

#### Training

Training CNN is divided into two stages to select a set of hyper-parameters: forward and backward propagation. In the forward propagation phase, CNN extracts a sample named *X*, and the label is $Y_{m}$ from the sample set, and inputting it into networks. The information is transferred from the input layer to the feature output layer through a stepwise transformation, and the corresponding actual output $O_{m}$ is calculated as follows:


(3) $$O_{m}=f_{n}\left(\ldots \left(f_{2}\left(f_{1}\left(XW_{1}\right)W_{2}\right)\ldots \right)W_{n}\right)$$

where *f* ( ) is an activation function and $W_{i}\left(i=1,2,\ldots ,n\right)$ is a trained mapping weight matrix.

The working principle of the convolutional layer and pooling layer with different layers is basically the same, but as the number of layers is deepened, the extracted feature maps are more abstract and more discriminative; however, too many layers may lead to issues, such as overfitting. After completing the convolutional mapping, CNN adjusts the output through the activation function and regards the features extracted by the convolutional layer as a function to perform nonlinear mapping. Commonly used activation functions are saturated nonlinear functions such as sigmoid and tanh. The unsaturated nonlinear function ReLU has been widely used in recent years. When the training gradient is degraded, ReLU converges faster than the traditional saturated nonlinear function, so the training speed is faster than the traditional method when training the whole networks. The weighted sharing strategy of CNN reduces the complexity of the networks. At the same time, the same feature map uses the same convolution kernel for feature extraction, and different feature maps use different convolution kernels. The convolutional layer preserves different local features, and it makes the extracted features have rotational and translation invariance.

#### Classification

In traditional CNN models, the Softmax classifier is often used to classify the input sample into the predefined classes. Its input is the output of the last fully connected layer, and its output is a probability to show the input samples belonging to the given class.

### Global Average Pooling

**Figure 2:**
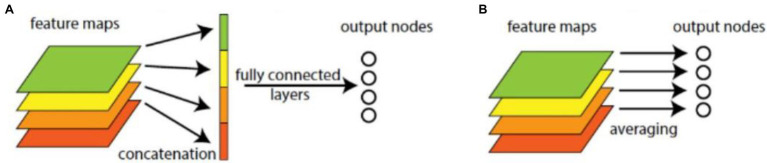
Fully connected and global average pooling (GAP) operations. **(A)** Fully connected operation. **(B)** GAP.

In common CNN models, through several convolutional layers and pooling layers, the learned features are input into several fully connected layers. The dense connection in fully connected layers makes it hard to interpret how the category level information from the objective cost layer feed back to the convolution layer. Furthermore, the fully connected layers are prone to overfitting and heavily depend on dropout regularization. The GAP operator is better than fully connected operator, as shown in Figure 2. It can reduce dimension and parameters, enhance the generalization ability, and overcome overfitting without optimizing the dropout parameters, because there is no parameter needed to optimize in the GAP layer. Let $x_{ij}^{k}$ be the *k*-th feature map with size *m* × *n* in the last convolutional layer, GAP is performed as follows,


(4) $$y_{\mathrm{GAP}}^{k}=\frac{1}{mn}\overset{m-1}{\underset{i=0}{\sum }}\overset{n-1}{\underset{j=0}{\sum }}x_{ij}^{k}$$

where $y_{\mathrm{GAP}}^{k}$ is output of the GAP layer.

### Inception

**Figure 3:**
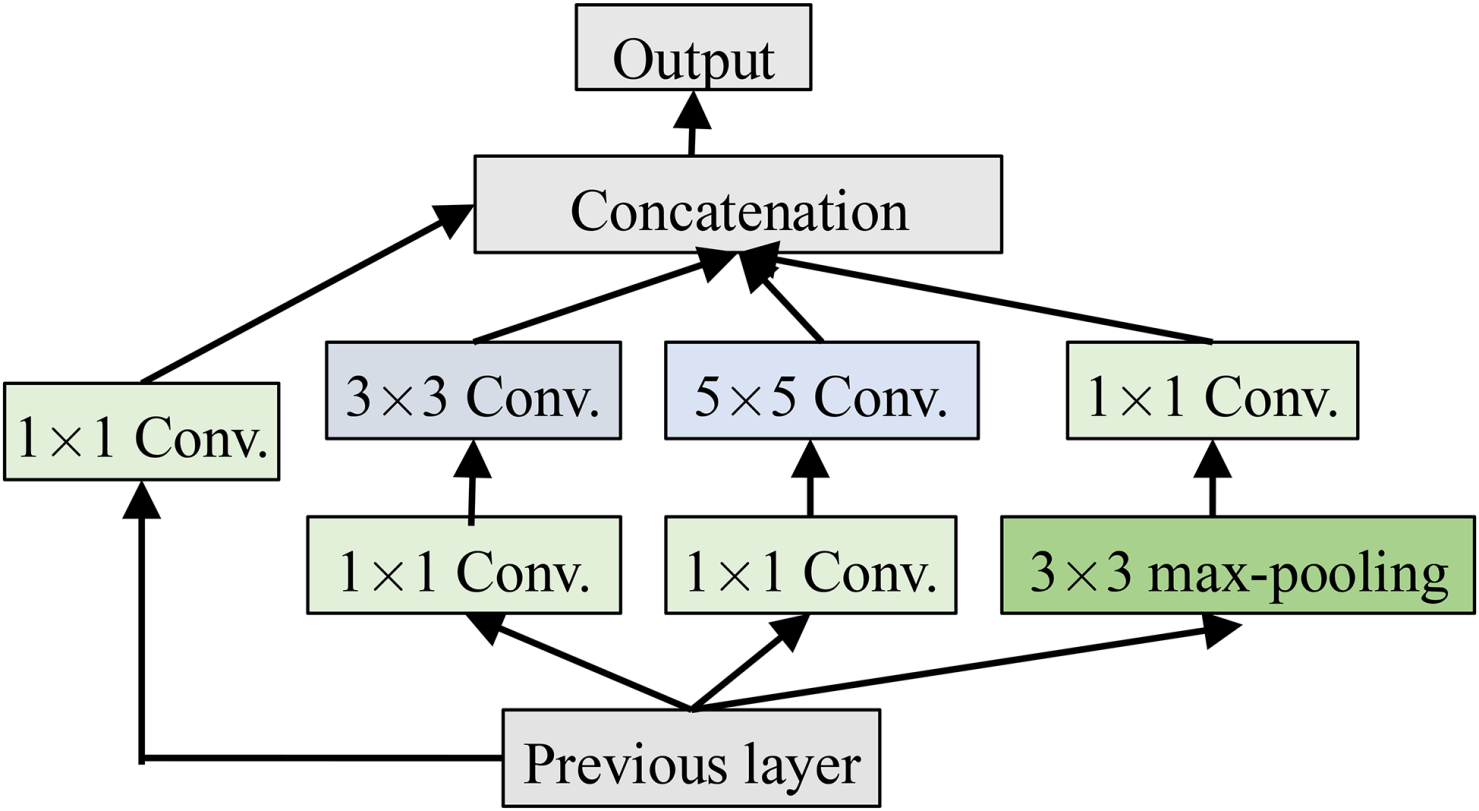
The structure of Inception.

Inception is a module in GoogleNet and has been validated to be better in the complex images classification tasks. It has multi-scale convolution kernels to extract the features of different scales from the input images by increasing the number of convolutional kernels and introducing multi-scale convolutional kernels. Inception is shown in Figure 3, where 1 × 1, 3 × 3, and 5 × 5 convolutional kernels are used to convolve the outputs of the upper layer at the same time to form a multi-branch structure. Feature maps obtained from the different branches are then concatenated to obtain different classification features of the input images. Processing these operations in parallel and combining all the results will result in better image representation. To make the feature map have the same size, each branch adopts the same padding mode, and the stride is 1. The 1 × 1 convolution operation is used, respectively, before 3 × 3 and 5 × 5 and after Max-pooling to reduce the amount of calculation.

### Softmax and Modified Softmax

Softmax maps the output of many neurons into (0,1) to implement the multi-classification task, which is calculated as follows,


(5) $$\begin{array}{l} {\mathrm{softmax}}_{j}\left(x^{\left(i\right)}\right)=p\left(c^{\left(i\right)}=j\left|x^{\left(i\right)},\theta \right.\right)\\ \;=\frac{\exp\left(\theta_{j}^{T}x^{\left(i\right)}\right)}{\sum_{i=1}^{C}\exp\left(\theta_{i}^{T}x^{\left(i\right)}\right)} \end{array}$$

where ${\mathrm{softmax}}_{j}\left(x^{\left(i\right)}\right)$ is the probability that $x^{\left(i\right)}$ belongs to the *j*-th class, $c^{\left(i\right)}$ is the label of $x^{\left(i\right)}$ , j=1,2,…,C,i=1,2,…,n and $\theta_{1},\theta_{2},\ldots ,\theta_{C}$ are the parameter of the classifier, and θ is a parameter matrix constructed by $\theta_{1},\theta_{2},\ldots ,\theta_{C}$ .

Generally, the gradient descent optimization algorithm is adopted to minimize the cost function of the Softmax classifier by partial derivatives, that is, the gradient of the cost function to the weight of the parameters can be obtained by calculating the partial derivatives. The loss function is calculated as follows,


(6) $$J\left(\theta \right)=\frac{1}{m}\overset{m}{\underset{i=1}{\sum }}\left(\begin{array}{l} -y^{\left(i\right)}\log\left(h_{\theta }\left(x^{\left(i\right)}\right)\right)\\ -\left(1-y^{\left(i\right)}\right)\log\left(1-h_{\theta }\left(x^{\left(i\right)}\right)\right) \end{array}\right)$$

where $y^{\left(i\right)}$ is the class label of $x^{\left(i\right)}$ , $h_{\theta }$ is defined as


(7) $$h_{\theta }\left(x^{\left(i\right)}\right)=\left(\begin{array}{c} p\left(y^{\left(i\right)}=1|x^{\left(i\right)};\theta \right)\\ p\left(y^{\left(i\right)}=2|x^{\left(i\right)};\theta \right)\\ \vdots \\ p\left(y^{\left(i\right)}=k|x^{\left(i\right)};\theta \right) \end{array}\right)=\frac{1}{\sum_{j=1}^{m}e^{\theta_{j}^{T}x^{\left(i\right)}}}\left(\begin{array}{c} e^{\theta_{1}^{T}x^{\left(i\right)}}\\ e^{\theta_{2}^{T}x^{\left(i\right)}}\\ \vdots \\ e^{\theta_{k}^{T}x^{\left(i\right)}} \end{array}\right)$$

Then θ is updated by


(8) $$\theta_{j}:=\theta_{j}-\alpha \nabla_{\theta_{j}}J\left(\theta \right),j=1,2,\cdots ,k$$

where α is a learn rate.

When Softmax regression solves model parameter θ , there is a “redundant” parameter set. According to Eq. (5), subtracting the vector ϖ from parameter vector $\theta_{j}$ does not affect the prediction result. Then, Softmax can be improved. The loss function of the modified Softmax is calculated as follows


(9) $$\begin{array}{l} J\left(\theta \right)=-\frac{1}{m}\left(\overset{m}{\underset{i=1}{\sum }}\overset{k}{\underset{j=1}{\sum }}1\left\{y^{\left(i\right)}=j\right\}\log p\left(y^{\left(i\right)}=j\mathrm{|;}x^{\left(i\right)}\mathrm{|;}\theta \right)\right)\\ +\frac{\lambda }{2}\overset{m}{\underset{i=1}{\sum }}\overset{n}{\underset{j=0}{\sum }}\theta_{ij}^{2} \end{array}$$

where λ>0 is a weight attenuation parameter, the cost function becomes strictly convex by adding $\frac{\lambda }{2}\overset{m}{\underset{i=1}{\sum }}\overset{n}{\underset{j=0}{\sum }}\theta_{ij}^{2}$ , so that a unique solution is guaranteed.

To solve the model parameter θ , the loss function J(θ) is minimized and the partial derivative of J(θ) is computed as follows,


(10) $$\nabla_{\theta_{j}}J\left(\theta \right)=-\frac{1}{m}\left(\overset{m}{\underset{i=1}{\sum }}\left(x^{\left(i\right)}\right)\left(\begin{array}{l} 1\left\{y^{\left(i\right)}=j\right\}\\ -p\left(y^{\left(i\right)}=j\mathrm{|;}x^{\left(i\right)}\mathrm{|;}\theta \right) \end{array}\right)\right)+\lambda \theta$$

Finally, the gradient parameter is updated by Eq. (8), and the parameter θ of Softmax is obtained.

## Modified CNN for Crop Disease Recognition

**Figure 4:**
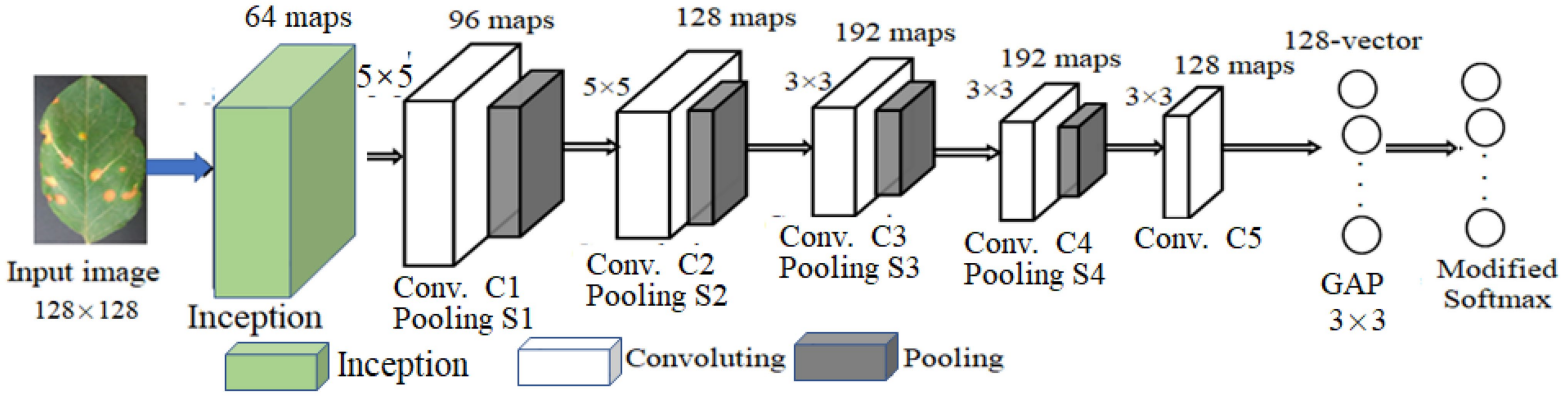
Architecture of modified convolutional neural networks (MCNN).

**Figure 5:**
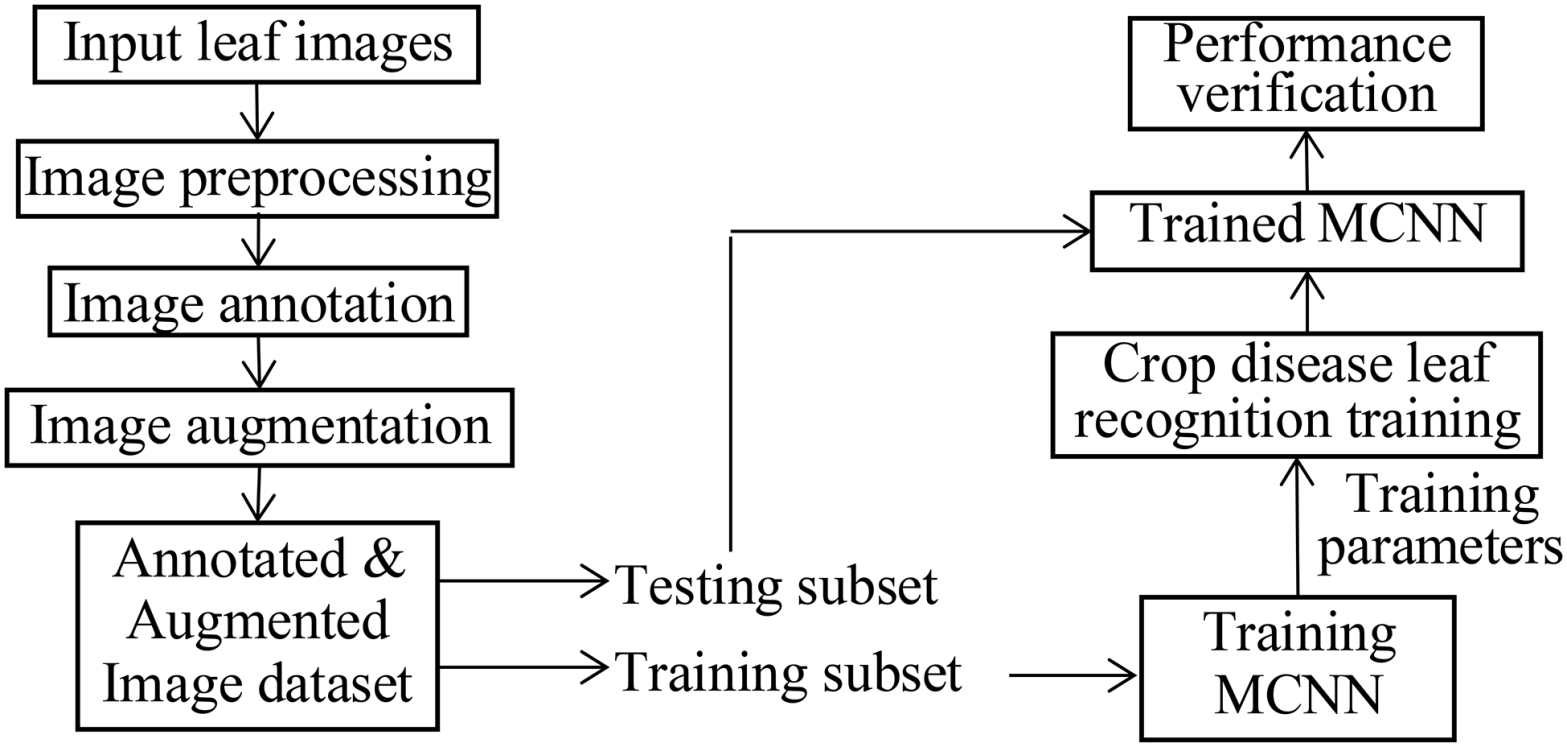
Framework of the proposed method.

LeNet5 only utilizes the deep convolutional feature information to build the classifier and indirectly loses the detailed texture information of the shallow convolutional layer. As for the problem of crop recognition, based on the idea of multi-layer feature fusion, a recognition algorithm of crop disease based on multi-layer feature fusion convolutional neural network is proposed (Zhang et al., 2020). Based on LeNet5, a modified CNN model (MCNN) is constructed to enhance the representation of the model to the image. Inception is introduced into MCNN. It has multi-scale convolutional kernels in different branches, and multi-scale image features are extracted by different receptive fields in each branch, which increases the width of the network and the adaptability of the network to lesion scale. The model includes one input layer, an Inception module, five convolution layers (C1, C2, C3, C4, and C5), four pooling layers (S1, S2, S3, and S4), a concatenation layer after Inception, global average pooling (GAP) layer, and one classification layer (i.e., modified Softmax). Its architecture is shown in Figure 4. Its parameters are given in Figure 4. Based on MCNN, a crop disease recognition method is proposed. Its framework is shown in Figure 5.

The steps of the proposed method are described as follows, where the training process of the MCNN model includes forward propagation and back propagation (Jg et al., 2021; Kundu et al., 2021):

1. Pre-treating the leaf image of crop disease. Since the disease image collected by the Internet of Things (IoT) Sensor has strong noise interference, we use a simple visual saliency algorithm to decompose multiple feature channels and multi-scales of the apple disease image, and then filter it, and a feature map is obtained. The feature map is fused to generate a visual saliency map. The center of the significant map area is determined. Taking the center of the significant map area as the spot center, a rectangular area is intercepted in the original disease image, and then, the K-means clustering method is used to cluster the significant areas to obtain the lesion image. Due to the complex diversity of disease images, the image preprocessing process is more complicated and time-consuming. Therefore, we try to extract a region of interest of the disease image as the input of the recognition method, omitting the process of background separation and the disease segmentation.
2. The RGB images are, respectively, input into MCNN to extract the features and recognize the image classes.
3. Training process of MCNN. In the training process, when the objective function is closer to the optimal value, the training is performed with a smaller learning rate than before, and the classification performance of the MCNN is improved by adjusting the parameters. The back-transfer calculation gradient is used to optimize the parameters of the MCNN to obtain better classification features. The nonlinear ReLU function is selected as the activation function to train the data after the convolutional neural layer.

The strengths and weaknesses of networks model training are mainly determined by the loss function. The lower the loss value of training and test, the better the model is trained, so that the whole networks can converge during the training phase. Assuming that each component image of the lesion image is the size of 128 × 128 two-dimensional feature map, the first convolution layer is Inception module with four branches. The first branch carries out 1 × 1 convolution of the input, which can carry out cross-channel feature transformation and improve the expression ability of the network. Meanwhile, it can raise and reduce the dimension of the output channel. The second branch uses 1 × 1 convolution first, and then joins 3 × 3 convolution, which is equivalent to two feature transformations. The third branch is the convolution of 1 by 1, and then the convolution of 5 by 5. The fourth branch is 1 × 1 convolution directly after 3 × 3 maximum pooling. The four branches of Inception Module are finally merged by an aggregation operation (aggregated on the dimension of the number of output channels).

The second convolution layer (C1) is convolved with a 64 × 64 size image through a 5 × 5 size convolution kernel. Twelve two-dimensional feature maps of 60 × 60 size were obtained. Among them, the same feature graph uses the same convolution kernels of 5 × 5. The number of parameters that C1 needs to train is 12 × (5 × 5 + 1) = 312, and the number of connections between the input layer and C1 is 312 × (60 × 60) = 1,123,200. In the first pooling layer (S1), 12 30 × 30 size feature maps are obtained by the activation function of equation (1), that is, by summing all the 2 × 2 sub-blocks X in C1 that do not overlap each other, and multiply by a weight *W*, plus an offset term b. Since the feature map size in C1 is 60 × 60, the obtained sub-sampling result is a feature subgraph of 30 × 30. Then, each sub-sampling layer uses a scaling factor of 2, whose purpose is to control the speed at which the zoom is degraded, because scaling is exponential scaling, and shrinking too fast also means that the extracted image features are coarser and will lose more image detail features. Each sub-sampling feature map needs to train two parameters, so S1 needs to train 12 × 2 = 24 parameters.

The third to sixth convolutional layers (C2–C5) is similar to C1. C2 also uses a convolution kernel of size 5 × 5, and the resulting feature map size is 26 × 26. After C1 and S1, the receptive field covered by each neuron of S1 is equivalent to 10 × 10 of the original image (the convolution kernel of C1 is 5 × 5, and the sampling sub-block size of the second pooled layer (S2) is 2 × 2, then 5 × 5 × 2 × 2 = 10 × 10). C2 extracts the characteristics of S1 through a 5 × 5-sized convolution kernel, and its receptive field is further expanded, which is equivalent to 50 × 50 of the original image. C1 obtains 12 mapping planes through one picture of the input layer. Now C2 needs to map 24 feature maps from 12 feature maps of S1. Here, certain skills are needed. When each feature map in C2 is convoluted, several feature maps or all feature maps in S1 are combined into an input and then convoluted. The data processing of the remaining convolutional layer and sub-sampling layer is basically the same as the previous layer. After multiple layers of convolution and down-sampling, the extracted features are more abstract and more expressive.

In GAP layer, 128 feature maps extracted by C5 are transformed into a 128-dimension vector, which is input into the modified Softmax classifier. The modified Softmax classifier is trained to obtain the trained model of apple disease identification. The test images are input the trained MCNN for model performance testing.

The training process of MCNN is divided into two stages: forward propagation and back propagation.

Step 1: Initialize the network weights.

Step 2: The input data are propagated forward through the convolution layer, down-sampling layer, and global pooling layer to obtain the output value.

Step 3: Calculate the error between the output value of the network and the target value.

Step 4: When the error is greater than our expected value, the error is sent back to the network, and the errors of the global pooling layer, the lower sampling layer, and the convolution layer are obtained successively. The error of each layer can be understood as the total error of the network, how much the network should bear. When the error is equal to or less than our expected value, we end the training.

Step 5: Update the weight according to the obtained error. Then, move on to the step 2.

## Experimental Results and Analysis

To validate the effectiveness of the proposed method, a number of plant disease recognition experiments are carried on two diseased leaf image databases of apple, namely, YangLing and Kaggle, and compared with Plant leaf disease recognition using SVM and PHOG Descriptor (SVMPHOG; Zhang and Sha, 2013), Plant leaf disease recognition using K-means-based segmentation and neural networks (KSNN; Bashish et al., 2011), Plant leaf disease recognition using image recognition technology (IRT; Qin et al., 2016), AlexNet (Jg et al., 2021), LeNet5 (Qi et al., 2021), VGG-16 (Dhaka et al., 2021), and CNN (Yadav et al., 2020). SVMPHOG, KSNN, and IRT are three feature extraction-based methods, where each leaf image is firstly segmented and their recognition rates rely heavily on the image segmented and feature extraction. AlexNet, LeNet5, VGG-16, and CNN are four kinds of widely used models. The parameters of the pre-training network in the constructed MCNN are set as: the number of training iterations 3,000, the number of samples processed per time Bsize = 128, weight decay is 0.0005, momentum is 0.9, and the initial learning rate is 0.001, which is reduced to half of the original value every 1,000 steps (Yadav et al., 2020), and Adam is as optimizer (Kingma and Ba, 2017). To verify the effectiveness of the proposed algorithm, the image recognition accuracy and average processing time per image are used to evaluate the algorithm. Image recognition accuracy refers to the ratio of the number of correct identification of all test samples to the number of test samples. Average processing time per image refers to the average processing time per image after the algorithm processes 100 images. The experimental conditions are CPU: Intel Corei3-2,120, 8G and Windows 64-bit, Ubuntu 16.04 LTS 64-bit system, Caffe deep learning open source framework, and Python as programming language. The computer has 16GB memory and Intel Core i7-6700KCPU @ 4.00 GHz X8 processor, Nvidia GTX980Ti graphics card.

The leaf image preprocessing is consistent with the traditional CNN model. The input images of the modified CNN model do not need to be pretreated with complex operations (such as segmenting diseased spots) and design features. Only the input of the original image can be used to acquire features layer by layer through back propagation. Since there is no publicly available apple disease image database, and the deep learning model requires a large number of samples for model training, to improve the adaptability and robustness of the model on the test set, it is necessary to expand the training set samples. We simulate the imaging conditions of the Internet of Things monitoring video in various environments by rotation, color and brightness changes, size scaling, contrast, saturation adjustment, adding noise and blur, etc. To reduce the number of training rounds, this study selected the offline augmentation of the training set samples, rotated the samples at four angles (0°, 90°, 180°, and 270°), and reversed them horizontally and vertically in all directions. We expand each image into 50 images, with a total of 450 × 50 = 22,500 training samples, then extract a region of interest containing most of the lesions of each image, and then cut each image into the 64 × 64 × 3 sized, and then the image segmentation method combined with the Itti algorithm and the K-means clustering method is used to segment each disease image to obtain the corresponding lesion image (Chen et al., 2014). Itti is widely used image saliency detection algorithm. It can detect the salient area for some given scene in the fields of computer vision. Since the image size and aspect ratio in the data set are inconsistent, the image is filled with edges, supplemented with average pixels as a square, and then scaled to the size of pixels to be stored in the hard disk.

### YangLing Dataset

All diseased leaves in two datasets were collected from the agricultural demonstration district of YangLing, Shanxi Province, China. The database contains 450 diseased leaf images of three kinds of disease: Alternaria leaf spot, Mosaic and Rust, and 150 leaf images per disease with typical symptoms were picked in fields and taken to laboratory, and expanded as flat as possible with a simple gray background.

**Figure 6:**
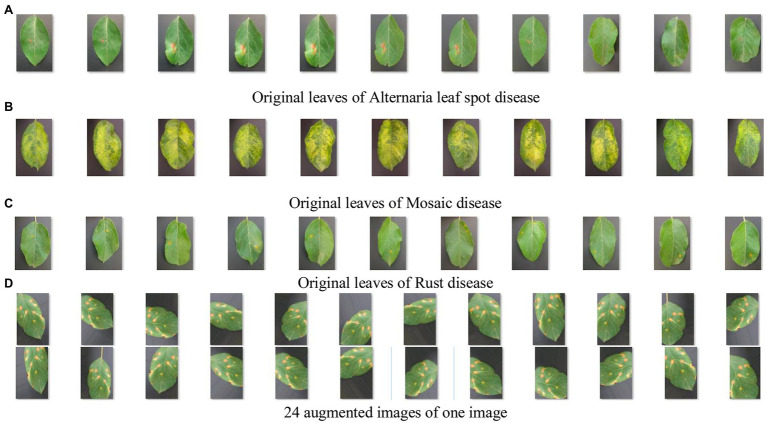
Apple diseased leaf images and augmented images. **(A)** Original leaves of Alternaria leaf spot disease. **(B)** Original leaves of Mosaic disease. **(C)** Original leaves of Rust disease. **(D)** Twenty-four augmented images of one image.

**Figure 7:**
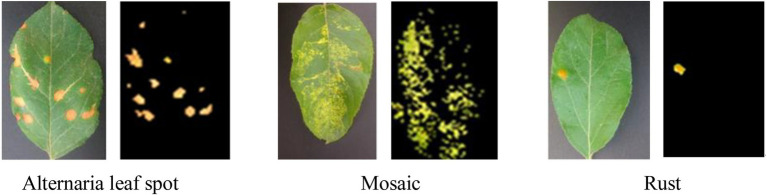
Apple diseased leaf images and corresponding segmented lesion images.

Figure 6 shows apple diseased leaf images and augmented images of YangLing dataset. Figure 7 shows three diseased leaves and their segmented lesions for the traditional disease recognition methods.

**Figure 8:**
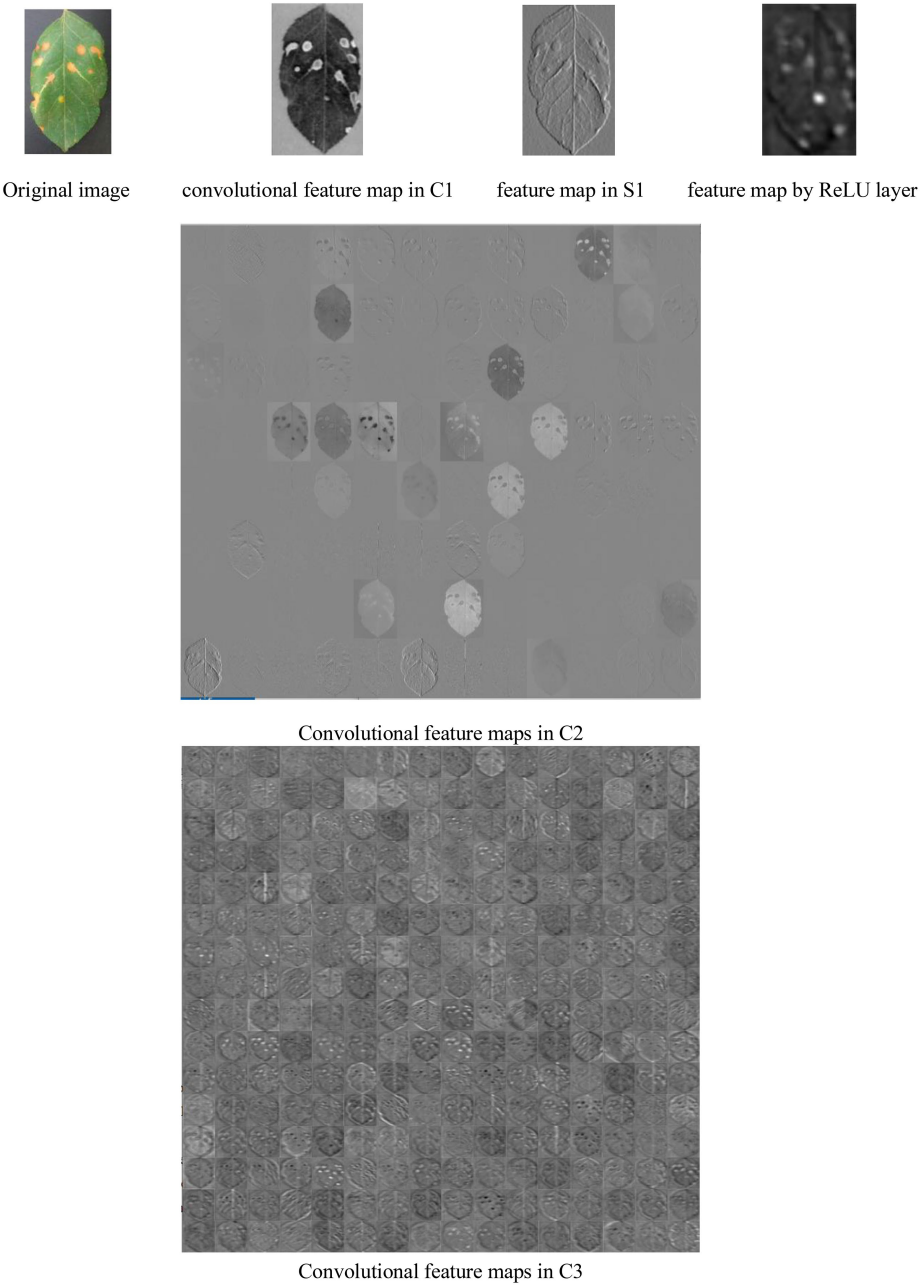
Feature images of a diseased RGB image in different layers.

Figure 8 shows a lot of convolutional feature maps. From Figure 6, it is found that MCNN can extract the high-level features of the diseased leaves. It is effective for disease recognition.

From Figure 8, it is seen that (1) the extracted feature maps by a lot of convolutional, pooling, and ReLU layers clearly retain the characteristic description corresponding the local areas of the input image, and notably different layers of MCNNs can extract the feature maps with the various properties. (2) The convolutional layers generate a large number of activation graphs (i.e., feature maps) with hundreds of convolutional kernels to capture the various features of the image. (3) Low-level feature maps contain low-level information such as texture and shape, while high-level feature maps can get more abstract semantic features by continuously combining low-level features. (4) In the migration process of the feature maps, the high-level feature maps are related to the pre-trained data set.

Several training-validation partitions are used in disease recognition experiments, such as 80/20, that is, the entire database is initially divided into training set and testing set by randomly splitting the all images, where 80% of them as training set, and 20% as testing set. For the training of the CNN model, the training images are used, while the rest images are kept for testing the performance of the models in classifying new, previously “unseen” images. We compare the recognition performance of the proposed method with seven leaf-based plant disease recognition methods/Plant leaf disease recognition using three traditional methods SVMPHOG, KSNN, and IRT and four CNN-based methods AlexNet, LeNet5, VGG-16, and CNN. In three traditional methods, the original images are needed to segment to obtain the spot images for disease recognition, while the proposed method uses the original images directly for disease recognition.

**Figure 9:**
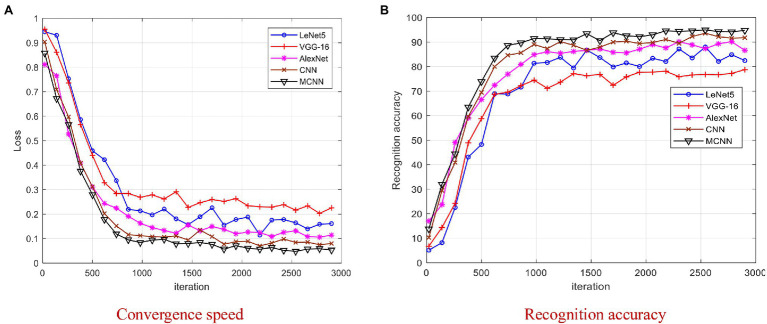
Comparison of four models on YangLing dataset. **(A)** Convergence speed. **(B)** Recognition accuracy.

The 80/20 splitting ratio of training-testing datasets is the most commonly used one in pattern recognition applications. To validate the convergence performance of MCNN, we compare MCNN with four models including AlexNet, LeNet5, VGG-16, and CNN. The Loss and recognition accuracies versus iterations on 80/20 splitting ratio are shown in Figure 9.

From Figure 9, it is found that MCNN outperformances the other models and achieves the best results after 2,000 iterations, because it is a multi-scale CNN and modified Softmax is used to recognize images. The training process of LeNet5 and VGG-16 is unstable due to the small disease leaf image dataset, and they cannot effectively extract the high-level features, which is difficult to meet the requirements of complex disease leaf image classification. From Figure 9, it is found that all models converge after 3,000 iterations. To be fair, we chose the trained model after 3,000 iterations for model testing.

We analyze the effectiveness of MCNN on various training-testing divisions versus: 50/50, 60/40, 70/30, 80/20, and 90/10, and compare it with CNN (Yadav et al., 2020). MCNN adopts modified while CNN adopts Softmax. The accuracies of MCNN and CNN on original and augmented datasets are shown in Table 1, respectively.

**Table 1:** Recognition results of MCNN and CNN on various training-testing divisions.

Accuracy	Division				
	50/50	60/40	70/30	80/20	90/10
CNN on original dataset	76.35	78.54	82.20	84.36	85.30
CNN on augmented dataset	89.14	90.21	91.44	92.19	93.12
MCNN on original dataset	91.41	92.04	93.14	93.83	94.17
MCNN on augmented dataset	92.67	93.38	93.76	94.21	94.24

From Table 1, it is seen that MCNN is better than CNN in all divisions even with the small training dataset, the accuracy of MCNN increases with increasing the training samples and modified Softmax is better than Softmax. This corresponds to a fact that the MCNN and CNN-based recognition approaches need a certain number of samples to train. From Table 1, it is also found that there is little difference in the recognition rate between 80/20 and 90/10. Therefore, in the following experiments, the 80/20 division and modified Softmax are adopted in MCNN.

After repeating the above 80/20 experiment 50 times, the average recognition accuracy and training time of seven kinds of the apple diseases are shown in Table 2.

**Table 2:** The average recognition accuracy and training time of apple disease on YangLing dataset by seven methods.

Results	Methods							
	SVMPHOG	KSNN	IRT	AlexNet	LeNet5	VGG-16	CNN	MCNN
Accuracy (%)	75.87	80.62	80.83	85.61	89.32	89.14	92.20	94.22
Training time (H)	0.04	0.06	0.01	12.32	8.54	11.08	12.25	8.15

### Kaggle Dataset

**Figure 10:**
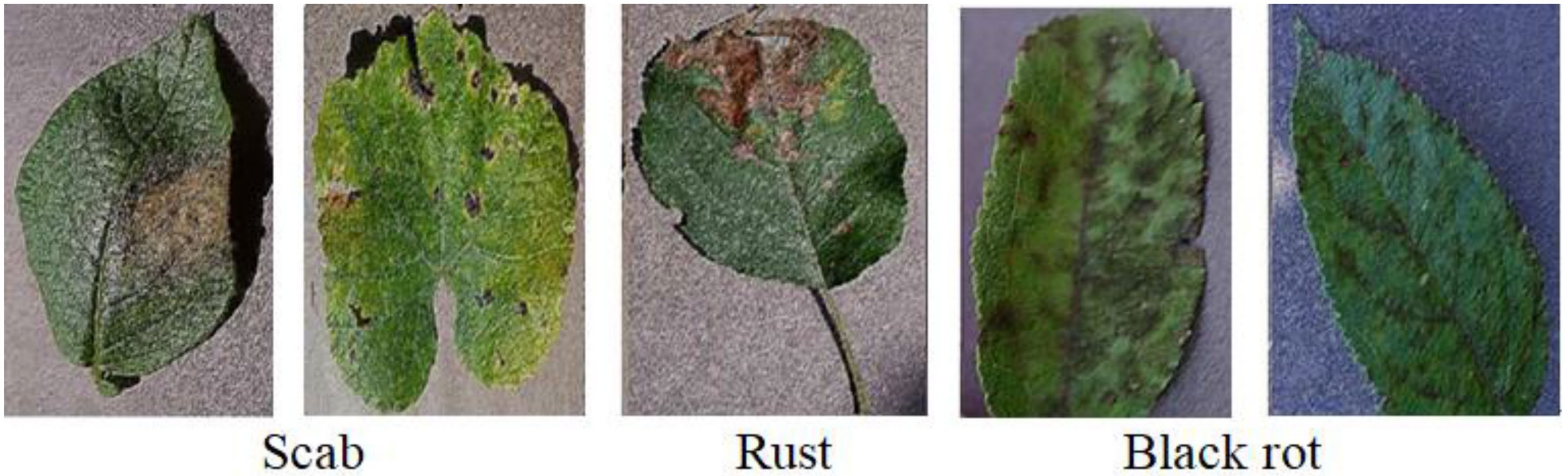
Apple disease leaf images.

The dataset is composed of four types of healthy and unhealthy apple leaves downloaded from Kaggle,^1^ consisting of 400 images, where 200 images are healthy leaf samples and the rest consists of three types of diseased leaf samples namely, Apple scab, Black rot, and Apple rust. Figure 10 shows some sample of images in the dataset. Each image is augmented by rotation, reversion, and reshape and segmented by Itti algorithm and K-means clustering method. In this dataset, each image is augmented to 10 samples and an augmented dataset containing 4,400 samples is constructed.

We validate MCNN on the Kaggle dataset and implement 80/20 experiment 50 times. The experiment configurations and parameter setting are similar to that on the YangLing dataset. The correct recognition rates of seven methods are shown in Table 3.

**Table 3:** The average recognition accuracy and training time of apple disease on Kaggle dataset by seven methods.

Results	Methods							
	SVMPHOG	KSNN	IRT	AlexNet	LeNet5	VGG-16	CNN	MCNN
Accuracy (%)	78.15	80.47	81.05	91.34	91.25	90.38	92.52	95.31
Training time (H)	0.03	0.05	0.01	11.13	8.44	10.68	9.75	9.12

From Figure 9 and Tables 1–3, it is found that MCNN outperforms the other seven models in accuracy and training time. The reasons may the multi-scale convolution module Inception is input into CNN to extract multi-scale lesion images, modified Softmax is adopted to classify apple disease leaf images, and GPA is used to speedup training. The classification accuracy of the five deep learning methods is much higher than that of three traditional methods, due to deep learning can learn features automatically from the complex disease leaf images. These features allow the various disease leaf images to be described at a higher level of abstraction while reducing the overfitting inherent in complex and deep networks. The training time of three traditional methods is very shorter than that of five deep learning models, because three traditional methods have few parameters in the classifier, while five deep learning models have more than thousands of parameters to be training by larger number of training samples. Although MCNN has an extra module Inception module comparing with LeNet5, its training time is litter than that of LeNet5, because MCNN uses GPA instead of fully connected layers in LeNet5, and the fully connected layers have about 80% training parameters in CNN, while GPA has none training parameters.

## Conclusion

The traditional crop disease identification method artificially extracts the classification features from the diseased leaf images, and then uses the classifier to classify and identify the disease types. Therefore, these methods, to some extent, are blind with a lot of complicated operation, low classification accuracy, and weak generalization ability. CNN can overcome these shortcomings. It can automatically learn the feature representations from raw image data but require enough labeled data to obtain a good generalization performance. As for the problem of apple disease identification, an apple disease identification method is proposed based on modified CNN (MCNN). This method can directly use the leaf images of apple disease to identify apple disease types. The experimental results show that the proposed method is effective and feasible.

Theoretically, through transfer learning, the model constructed in this paper can be transferred to other crop disease identification tasks but requires fine-tuning. MCNN is a lightweight multi-scale CNN, which is easy to implement on a general PC and can be applied to crop disease recognition system. However, it cannot be applied to the crop disease recognition system based on IoT, because the background of the data set of the method in this paper is relatively simple. The image background collected by the IoT is very complex. With the development of Internet of things (IoT) applications, the leaf disease can be detected and recognized by IoT from the fields, but it is a challenging task, because the collected images by IoT contain a lot of background and noise, and the disease spot features may be submerged in the image, which is not easy to detect and recognize. Therefore, firstly, the collected leaf image should be segmented from the complex background. However, it is still difficult to segment disease leaf images from complex field background. The future work is proposed an effective image segmentation algorithm to segment the collected image by IoT.

## Data Availability Statement

The original contributions presented in the study are included in the article/supplementary material; further inquiries can be directed to the corresponding author.

## Author Contributions

PL: original draft preparation, methodology, writing, and experiments. XS: data collection, pre-processing, and model verification. RJ: experimental supplement and later paper revision. All authors contributed to the article and approved the submitted version.

## Funding

This work is supported by the Guiding Plan of 2022 Key Scientific Research Projects of Henan Universities, no. 22B520049 and the Science and Technology Project of Henan Provincial Education Department nos. 212102210404 and 212102210406.

## Conflict of Interest

The authors declare that the research was conducted in the absence of any commercial or financial relationships that could be construed as a potential conflict of interest.

## Publisher’s Note

All claims expressed in this article are solely those of the authors and do not necessarily represent those of their affiliated organizations, or those of the publisher, the editors and the reviewers. Any product that may be evaluated in this article, or claim that may be made by its manufacturer, is not guaranteed or endorsed by the publisher.

## Footnotes

^1^ https://www.kaggle.com
